# Utility and real-world clinical outcomes of next-generation sequencing in advanced non-small-cell lung cancer in the South Indian population

**DOI:** 10.1093/oncolo/oyaf168

**Published:** 2025-07-31

**Authors:** Thattungal Manoharan Anoop, Lakshmi Raj, Pallavi Nair, Athira Vincent

**Affiliations:** Additional Professor, Department of Medical Oncology, Regional Cancer Centre, Thiruvananthapuram, India; Senior Resident, Department of Medical Oncology, Regional Cancer Centre, Thiruvananthapuram, India; Assistant Professor, Department of Medical Oncology, Regional Cancer Centre, Thiruvananthapuram, India; DM Resident, Department of Medical Oncology, Regional Cancer Centre, Thiruvananthapuram, India

**Keywords:** next-generation sequencing (NGS), advanced NSCLC, survival

## Abstract

**Background:**

Next-generation sequencing (NGS) is an advanced sequencing technology that enables rapid sequencing of numerous DNA strands and performs simultaneous analysis of various genes and diverse genomic characteristics. While there is a lack of substantial evidence available, the extent to which NGS may improve clinical outcomes among cancer patients in a real-world scenario remains uncertain.

**Objective:**

To investigate the clinical utility of NGS in patients treated with advanced non-small cell lung cancer (NSCLC) and its impact on real-world clinical outcomes, treated with targetable or non-targetable agents.

**Materials and methods:**

This was a prospective observational study conducted in 322 participants distributed over 2 broad categories- NGS and non-NGS. The NGS category consisted of patients who underwent genetic mutation screening by the NGS method. This group was further categorized into 2 subgroups-NGS—targetable and NGS-non-targetable. The non-NGS category consisted of patients who did not undergo mutation testing by the NGS method.

**Results:**

There was a significant difference in overall survival between NGS and non-NGS groups (*P* = .0038). There was a significant difference between NGS targetable and non-targetable groups in terms of progression-free survival (PFS; *P* = .0016) and overall survival (OS; *P* < .0001). There was a significant difference between NGS-matched and non-matched groups in terms of PFS (*P* < .00011) as well as OS (*P* < .0001).

**Conclusions:**

NGS significantly improved survival in advanced NSCLC. Patients who received treatments matched to their NGS results experienced significantly longer survival compared to those with non-matched treatments.

**Implications for Practice**

The treatment advances in precision medicine in lung cancer have led to improvements in patient outcomes. The use of Next-generation sequencing (NGS) for molecular profiling of genetic alterations plays a crucial role in the selection of appropriate targeted therapy. Despite the increased use of NGS in real clinical practice, there are limited data regarding the true influence of these tests on clinical outcomes in advanced non-small cell lung cancer (NSCLC). This study reveals the clinical effectiveness of NGS in advanced NSCLC patients and its impact on real-world clinical outcomes for those receiving either targetable or non-targetable therapies.

## Introduction

Non-small cell lung carcinoma (NSCLC) is the most prevalent form of lung cancer, representing approximately 75% of all cases.^1^ The routine identification of mutations in epidermal growth factor receptor (EGFR), ROS-1, and anaplastic lymphoma kinase (ALK) genes is a standard practice in NSCLC to tailor targeted therapies based on an individual’s specific genetic profiles.^2-5^ Around, 32.3% of NSCLC cases exhibit mutations in the EGFR gene, with alterations predominantly found in the tyrosine kinase domain, specifically the L858R mutation in exon 21 and deletions in exon 19 (exon19del), while 5% of NSCLC cases are associated with ALK rearrangements.^4,5^ Additionally, 0.9%-2.6% cases of NSCLCs are identified with ROS-1 rearrangement, with a higher occurrence in nonsmokers, women, and younger individuals.^6^

The National Comprehensive Cancer Network for immunotherapy recommends the detection of programmed death-ligand 1 (PD-L1) as the only prognostic biomarker for the development of effective therapies against metastatic NSCLC.^7^ The implementation of precision medicine, driven by a deeper understanding of the genomic alterations leading to the proliferation of cancer cells, has significantly transformed the therapeutic landscape for NSCLC.^8^ This includes the use of PDL-1 immune checkpoint inhibitors, viz. atezolizumab, duvalumab, and avelumab, which are reported to improve overall survival (OS) rates in cancer patients.^7^

The evaluation of tumor mutation burden in NSCLC patients through next-generation sequencing (NGS) serves as a valuable tool for predicting their response to immunosuppressive therapy.^9,10^ NGS is an advanced sequencing technology that enables rapid sequencing of numerous DNA strands and performs simultaneous analysis of various genes and diverse genomic characteristics, including single-nucleotide variants and copy number variations.^2^ While there is a lack of substantial evidence available, the extent to which NGS may improve clinical outcomes among cancer patients remains uncertain.

NGS provides a comprehensive view of the molecular landscape of NSCLC, deciphering tumor heterogeneity and revealing co-occurring or mutually exclusive genetic alterations. Identifying targetable mutations like EGFR and ALK are particularly significant in view of their significant activity in CNS penetration, and their use eventually leads to better survival.

In patients with advanced NSCLC, PFS for first-line treatment was significantly different among therapeutic strategies. The chemotherapy-treated patients showed the worst outcome, while the targeted therapy-treated patients exhibited the longest PFS, especially in cases of ALK and EGFR-positive NSCLC patients. Osimertinib, a third-generation *EGFR***-**TKI, seems to be more potent and more effective, in terms of survival, than chemotherapy or standard *EGFR*-TKIs in first-line treatment. Lorlatinib is a brain-penetrant, third-generation ALK TKI that has greater coverage of *ALK* resistance mutations than second-generation ALK inhibitors or chemotherapy. In this updated 5-year analysis from the CROWN study, lorlatinib continued to show superior efficacy over crizotinib with remarkable PFS benefit and intracranial efficacy. The PFS benefit, which exceeds 5 years, represents the longest reported PFS outcome with any molecular targeted therapy across metastatic solid tumors.

In real clinical practice, especially in India, PDL1 test reports are available weeks before the normal turnaround time for Mutational status results. Hence, physicians were tempted to initiate chemotherapy along with immunotherapy. Hence, some patients experience severe immune-related adverse events (pneumonitis and colitis) after osimertinib administration in patients who received EGFR-TKIs soon after treatment with PD-1 inhibitors. In view of this complication, Identification of the EGFR mutation is useful before initiation of immunotherapy. Clinicians should be aware of these risks before selecting and initiating EGFR-TKI therapy for up to 6 months after following prior PD-1/PD-L1 inhibitor use.

Genomic lung cancer data of Indian lung cancer patients is under-represented in available databases.

In a study by Pragya Gupta et al,^11^ clinicopathologic characteristics and mutational profiling data was analyzed in 154 cases of lung adenocarcinoma from the eastern part of India using next-generation sequencing. The most common mutated gene was TP53 gene (37.6%, n = 58) followed by EGFR (32.4%, n = 50), KRAS (18.18%, n = 28), ERBB2 (3.2%, n = 5), BRAF (1.94%, n = 3).

In a retrospective study from India by Shah, Minit et al,^12^ the clinical impact of NGS in 1230 Indian NSCLC patients has been minimally explored, and they identified driver-gene alterations in 64.8% cases, and almost 62.1% received targeted therapy based on NGS. With the receipt of targeted therapy, the median OS of driver-positive patients was 26.7 months compared to 9.3 months without driver mutations. Another study from India by Jha, Prerana et al ^13^ analyzed the genomic profiles of 325 lung adenocarcinoma and 81 lung squamous carcinoma samples from Indian patients using targeted sequencing of 50 cancer-related genes. They identified that alterations in *EGFR* (45.8%), *TP53* (27.4%), *ALK* (11.4%), and *KRAS* (10.2%) were predominant in adenocarcinoma. Squamous carcinoma exhibited prevalent alterations in *TP53* (40.7%), *PIK3CA* (17.3%), *CDKN2A* (8.6%), and a higher frequency of *EGFR* alterations (18.5%) in lung squamous carcinoma patients. This study emphasizes the importance of the clinical utility of NGS for routine diagnostics which revealed unique genomic variations of adenocarcinoma and squamous carcinoma patients, with significant indications for precision medicine and clinical practice of lung cancers. The utility of such short targeted NGS panels for identifying the most common alterations is particularly useful in clinical implications for personalizing medicine for patients could improve clinical accessibility and cost-effectiveness, especially in a low-resource country like India.

Therefore, in the present study, we examined the clinical effectiveness of NGS in advanced NSCLC patients and evaluated its impact on real-world clinical outcomes for those receiving either targetable or non-targetable therapies.

## Methods

### Study design

This was a prospective observational study conducted at the Lung Cancer Clinic of the Department of Medical Oncology, Regional Cancer Center, and Trivandrum. The study period spanned from January 2021 to January 2023.

### Inclusion criteria

All newly diagnosed patients between the ages of 18 to 70 years with metastatic NSCLC who were treated at the Lung Cancer Clinic in the Department of Medical Oncology and planned for first-line treatment were included in the study. All patients provided informed consent.

### Patient stratification and treatment protocols

Baseline clinical data for patients were collected, including age, gender, smoking history, pathological type, family cancer history, comorbidities, and initial stage at diagnosis, driver gene status, and details on the treatment given. All patients underwent NGS testing that had multi-gene panel testing, including the 9 genes as molecular biomarkers (EGFR, ALK, ROS1, KRAS, HER2, BRAF, MET, RET, and NTRK) mentioned in the National Comprehensive Cancer Network guidelines (10). Patients who underwent NGS testing were classified as the NGS group, whereas those who received conventional gene mutation testing were classified as the non-NGS group. Targeted therapy was administered to patients with identified oncogenic driver mutations based on NGS results, whereas standard chemotherapy or chemo-immunotherapy was provided to those without detectable targetable mutations.

### Study objectives

The study specifically aimed to compare survival outcomes between patients who underwent NGS testing (NGS group) and those who did not (Non-NGS group), as well as between those treated with targetable agents (NGS-Targetable group) and those treated with non-targetable agents (NGS-Non-Targetable group) based on NGS results. Therapeutic efficacy was evaluated by measuring progression-free survival (PFS) and overall survival (OS). PFS was defined as the time from the start of systemic treatment to the first occurrence of radiological or clinical disease progression or death from any cause. OS was defined as the time from the initiation of any systemic treatment to death from any cause.

### Statistical analysis

Descriptive statistics were used to describe the social and demographic profiles. All quantitative variables were expressed as median ± IQR and mean ± SD. Proportions as percentages were used to express categorical variables. Kaplan–Meier plots were used to estimate the loss or gain of function over the years. For the Statistical analysis, SPSS Version 25 was used, and R Software (R 4.1.1) at 95% CI and 80% power. A *P*-value of < .05 was considered significant.

## Results

This analysis consisted of 322 participants distributed over 2 broad categories- NGS and non-NGS. The NGS category consisted of 84 patients who underwent genetic mutation screening by the NGS method. This group was further categorized into 2 subgroups—targetable and NGS-non-targetable. The NGS-targetable group comprised 84 patients in which targetable agents were found through NGS. The NGS-non-targetable subgroup comprised 104 patients who underwent NGS, but no targetable agents were found. The non-NGS category consisted of 135 patients who did not undergo mutation testing by the NGS method. The baseline characteristics of the study participants have been outlined in **Table 1**.

**Table 1.** Baseline characteristics of the study participants (*N* = 322).

Characteristic	NGS targetable	NGS non targetable	Non-NGS
***n***	84	135	103
**Age**			
Median (years)	56	61	61
Mean (years)	56.8	59.8	59.1
**Gender**			
Male, *n* (%)	44 (51.2)	94 (69.6)	73 (70.9)
**Habits**			
Smoking, *n* (%)	23 (27.4)	75 (55.6)	64 (62.1)
Alcohol consumption, *n* (%)	14 (16.7)	49 (36.3)	NA
**PDL1 mutation status**			
<1%, *n* (%)	67 (79.8)	108 (80)	175 (79.9)
1%-49%, *n* (%)	12 (14.3)	20 (14.8)	32 (14.6)
>50%, *n* (%)	5 (6)	7 (5.2)	12 (5.5)
**Comorbidites**			
Hypertension, *n*	24	45	33
Diabetes Mellitus, *n*	28	45	25
Coronary artery disease, *n*	5	10	6
Asthma, *n*	4	12	6
None, *n*	30	45	46
*NA- data not recorded.*			

### Demographic analysis

#### Age

The overall mean age of patients was found to be 58.8 years in the study, with the majority population lying between the 60 and 70 years category. (**Supplementary Figure 1**) There was a significant difference observed in age between NGS targetable and NGS non-targetable groups *(P = .036).*

#### Gender

The majority of patients in this study were males in all 3 categories. The overall proportion of males in the study was 64.9%. (**Supplementary Figure 2**).

#### Smoking

We found that half of the study population comprised smokers (50.3%; **Supplementary Figure 3**).

#### PDL1 mutation status

In this study, most patients were negative for PD-L1 mutation (expression < 1%). Overall, 79.9% of patients tested negative for PD-L1, with each subcategory demonstrating more than 75% of cases being negative for PD-L1 mutation (**Supplementary Figure 4**).

#### Deaths

This study reported an overall mortality rate of 51.2%. However, the non-NGS group exhibited the highest number of fatalities at 79.9%.

### Survival analysis

#### NGS subgroup

**Figure 1.**
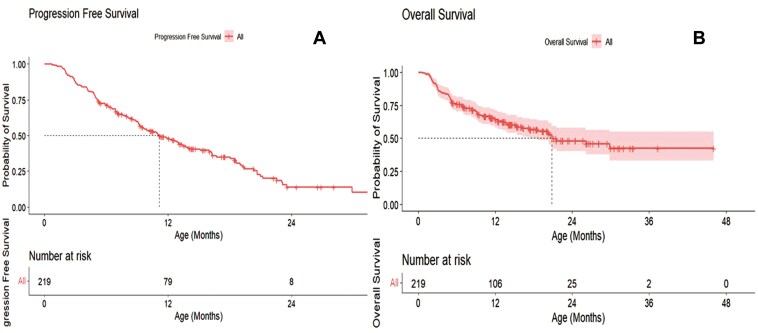
Survival analysis of NGS subgroup. Kaplan–Meier curves depicting survival curves for patients with the NGS subgroup. Progression-free survival for all patients with the NGS subgroup represented by A. Overall survival for all patients with the NGS subgroup represented by B.

This study reported a median PFS time of 11.1375 months with CI (9.4948, 13.700) and overall median survival time of 16.32 months with CI: (13.83, 20.27; **Figure 1A and B**), respectively.

#### Comparison (NGS vs non-NGS)

.

**Figure 2.**
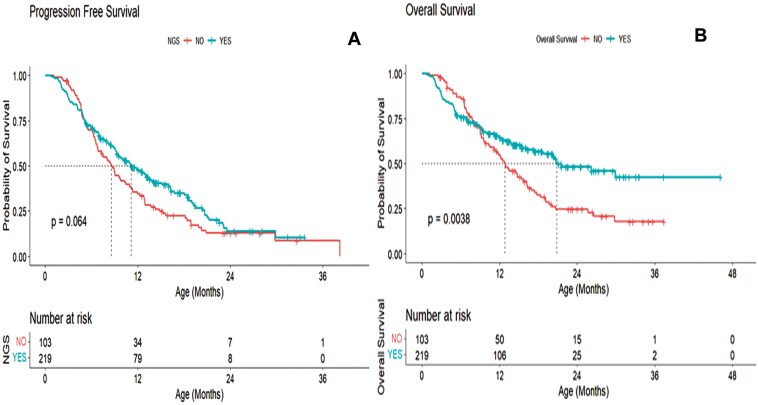
Survival analysis using Kaplan–Meier curves depicting survival curves for patients with NGS versus non-NGS subgroup. Progression-free survival is represented by A. Overall survival is represented by B.

The median PFS time in the NGS group was 11.13 months with CI: (9.49, 13.70), and that for the non-NGS group was 11.64 months with CI: (8.01, 6.20). There was no significant difference between the NGS and Non-NGS group in terms of PFS *(P = .064;***Figure 2A**).

The overall median survival time for the NGS group was 20.82 months with CI (11.13, 16.19) and that for non-NGS group was 12.81 months with CI: (17.37, NA). There was a significant difference between the overall survival of NGS versus Non NGS groups *(P = .0038;***Figure 2B**).

#### Comparison (NGS-targetable vs NGS-non-targetable)

**Figure 3.**
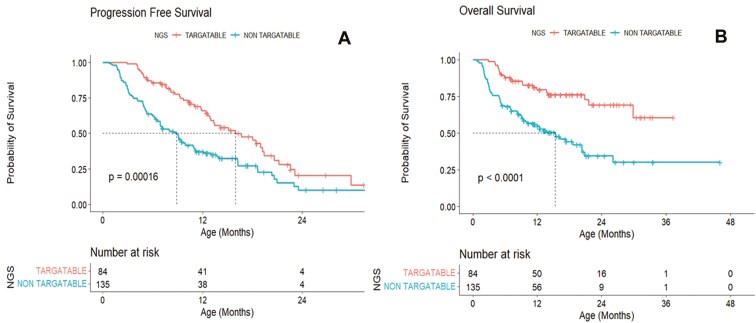
Survival analysis using Kaplan–Meier curves depicting survival curves for patients with NGS targetable versus NGS non-targetable. Progression-free survival is represented by A. Overall survival is represented by B.

The median PFS time in the NGS targetable group was 15.96 months with CI: (12.97, 20.63) and that for the NGS non-targetable group was 11.23 months with CI: (9.36, 15.11). There was a significant difference between NGS targetable and non-targetable groups in terms of PFS (*P* = .0016; **Figure 3A**).

The overall median survival time in the NGS targetable group was nil and that for NGS non-targetable group was 15.37 months with CI: (9.79, 20.82). There was a significant difference between NGS targetable and NGS non-targetable treatment groups (*P* < .0001; **Figure 3B**).

#### Comparison (PDL1 status)

The median PFS time for PDL1 expression (<1%) was 11.23 months with CI: (9.36, 15.11); for PDL1 expression (1 to 49%) was 13.3388 months with CI: (7.19, NA); for PDL1 expression (>50%) was 6.48 months with CI: (3.64, NA). There was no significant difference between the PDL1 mutation <1% and 1% to 49% groups in terms of PFS (**Supplementary Figure 5A**).

The overall median survival time for PDL1 expression (<1%) was 21.552 months with CI: (18.62, NA); for PDL1 expression (1% to 49%) was nil; or PDL1 expression (>50%) was 9.199 months with CI: (3.64, NA). There was no significant difference between PDL1 expression <1% and 1% to 49% groups in terms of OS (**Supplemental Figure 5B**).

#### Comparison (matched vs non-matched)

B.

**Figure 4.**
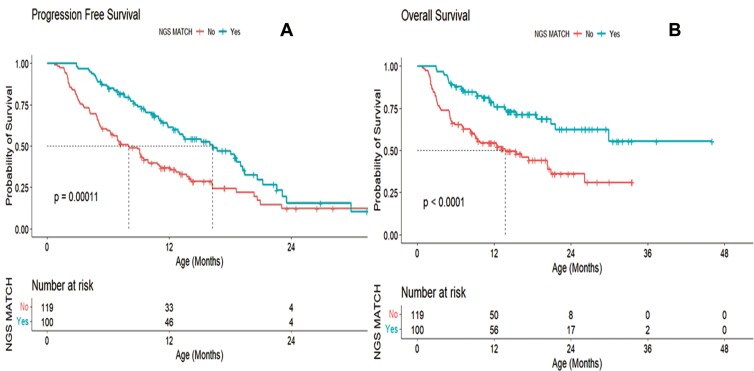
Survival analysis using Kaplan–Meier curves depicting survival curves for patients with NGS matched versus NGS non-matched. Progression-free survival is represented by A. Overall survival is represented by B.

The median PFS time for the NGS-matched group was 16.2628 months with CI: (12.84,19.41) and that for the NGS-non-matched group was 11.64 months with CI: (8.01,6.20). There was a significant difference in PFS between NGS-matched and non-matched groups *(P < .00011;***Figure 4A**).

The overall median follow-up time for the NGS-matched group was 16.42 months with CI: (14.58, 19.48) and that for the NGS-non matched group was 16.03 months with CI: (14.06, 19.41). There was a significant difference in OS between NGS-matched and non-matched groups *(P* < .0001; **Figure 4B**).

#### Multivariate Cox regression models and hazard ratio estimates

##### Progression-free survival

In the PFS analysis, Female sex demonstrated a borderline association with increased risk (HR = 1.415, 95% CI: 0.992-2.019, *P* = .056), suggesting a potential trend toward worse PFS in females (Table 2). PDL1 STATUS was the only variable showing a statistically significant association at the 5% level (HR = 2.356, 95% CI: 1.034-5.366, *P* = .041), indicating that positive PDL1 status is associated with more than twice the risk of disease progression. Similarly, 3 category PDL1STATUS (<1%, 1% to 49%, >50%) showed a near-significant protective effect (HR = 0.314, 95% CI: 0.094-1.054, *P* = .061), indicating a possible reduction in risk, though the result did not meet the 5% significance threshold. Non-NGS showed a nonsignificant trend toward increased risk (HR = 1.484, *P* = .123), while Match was also not significantly associated with PFS (HR = 0.714, *P* = .195). Mutation status had no meaningful association with PFS (HR = 1.006, *P* = .979). In summary, PDL1 positivity was significantly associated with worse PFS, while female sex toward borderline risk for worse PFS. Other variables were not significant predictors of PFS in this model.

**Table 2.** Multivariate Cox regression model and hazard ratio estimates of progression-free survival.

Variable	Coefficients	SE	Wald	df	Sig.	Exp(B)	95.0% CI for Exp(B)	
							Lower	Upper
SEX_Female	0.347	0.181	3.665	1	0.056	1.415	0.992	2.019
NGS(Non)	0.394	0.256	2.375	1	0.123	1.484	0.898	2.450
Match	−0.337	0.260	1.681	1	0.195	0.714	0.429	1.188
Three_cat_PDL1STATUS	−1.157	0.618	3.512	1	0.061	0.314	0.094	1.054
PDL1STATUS	0.857	0.420	4.164	1	0.041	2.356	1.034	5.366
Mutation status	0.006	0.238	0.001	1	0.979	1.006	0.631	1.606

##### Overall survival

Based on the analysis using a 5% significance level, only the variable Non-NGS showed a statistically significant association with the outcome (HR = 2.212, 95% CI: 1.127-4.340, *P* = .021), indicating that patients with Non-NGS had more than twice the hazard compared to the reference group (Table 3). This suggests Non-NGS is a strong and meaningful predictor of increased risk. The variable Female sex demonstrated a borderline association (HR = 1.588, 95% CI: 0.998-2.528, *P* = .051), indicating a possible trend toward increased risk in females, though it did not meet the strict threshold for significance. Mutation Status was associated with a nonsignificant trend toward reduced risk (HR = 0.619, 95% CI: 0.363-1.057, *P* = .079), suggesting a potential protective effect. However, both variables require cautious interpretation and may warrant further investigation in larger studies. The remaining variables, Match (*P* = .662) and PDL1STATUS (*P* = .241), were not significantly associated with the outcome and did not show meaningful trends. Their hazard ratios also had wide confidence intervals that included 1, indicating no evidence of effect. Overall, Non-NGS emerged as the only statistically significant predictor at the 5% level with a strong and meaningful predictor of increased risk, while other variables showed nonsignificant or borderline effects.

**Table 3.** Multivariate Cox regression model and hazard ratio estimates of overall survival.

Variable	Coefficents	SE	Wald	df	Sig.	Exp(B)	95.0% CI for Exp(B)	
							Lower	Upper
SEX_Female	0.463	0.237	3.803	1	0.051	1.588	0.998	2.528
NGS(Non)	0.794	0.344	5.324	1	0.021	2.212	1.127	4.340
Match	−0.143	0.327	0.192	1	0.662	0.867	0.456	1.646
Three_cat_PDL1STATUS	−0.302	0.697	0.187	1	0.665	0.740	0.189	2.897
PDL1STATUS	0.556	0.474	1.373	1	0.241	1.743	0.688	4.414
Mutation status	−0.479	0.272	3.091	1	0.079	0.619	0.363	1.057

## Discussion

In our study, 68% of the 322 advanced NSCLC patients underwent NGS testing, and a notable 38% of those received targeted therapy based on their NGS results. This highlights the effective use of NGS in guiding treatment decisions for a significant proportion of patients. In comparison, a larger cohort study of 5688 NSCLC patients by Presley CJ et al showed that while broad-based genomic sequencing was used in 15.4% of cases, only 4.5% of patients received targeted therapy.^14^ This contrast suggests that while NGS has the potential to guide treatment in a substantial number of patients, its real-world application may vary significantly across different settings or populations.

Our results indicate that a smaller proportion of our study population exhibited positive PD-L1 expression (20%). Few studies have reported similar findings. Cooper W.A. et al. found a PD-L1 positive expression rate of 7.4% among NSCLC patients^15^ while Kim M.-Y. et al. reported positive PD-L1 expression in 27% of cases^16^ and Konrad P. et al. reported a rate of 32.5%.^17^ Most of the existing literature contradicts our findings, reporting positive PD-L1 expression in approximately 50%-70% of cases.^18-20^ PD-L1 protein expression varies significantly across clinical studies on NSCLC. Furthermore, data regarding the relationship between PD-L1 expression and clinicopathological factors differ widely. Another plausible explanation for the variability in PD-L1 expression in NSCLC may be attributed to the use of different scoring methods and cutoff levels for evaluation. Variations in the percentage of patients expressing PD-L1 in NSCLC cells across studies could also stem from the use of different antibody clones (22C3, 22-8, SP142, and SP263).

We observed a significant increase in median OS for the NGS group compared to the non-NGS group (20.82 vs 12.81 months, *P = .0038*). In contrast, Tu T et al. reported that while NGS detection did not notably improve median OS compared to conventional gene detection, it did lead to a significantly higher 1-year survival rate (83.2% vs. 68.1%, *P* = .0015), indicating short-term survival benefits.^21^ Similarly, Kang DW et al. found no significant difference in OS or mortality risk between the NGS and single-gene testing groups after adjusting for various factors.^2^

Further analysis within the NGS group showed that patients treated with therapies based on NGS results had a significantly longer mean OS compared to those who did not receive targeted treatments (33.87 vs. 15.37 months, *P* < .0001). Similar findings were reported in other studies. Tu T et al. found that patients receiving targeted therapy had a significantly longer median OS compared to those without targeted therapy (28.3 vs. 15.4 months, HR = 0.5426, *P* < .0001), with results remaining consistent after propensity score matching.^21^ Additionally, Kris MG et al. tested tumors from 1007 lung adenocarcinoma patients and observed that those with genotype-directed therapies had a longer median survival (3.5 years) compared to those without targeted treatment (2.4 years), suggesting that targeting therapy based on genetic drivers can enhance survival.^22^

In our cohort, patients who received treatments matched to their NGS results experienced significantly longer median PFS and overall survival (OS) compared to those with non-matched treatments *(P* < .0001). Similarly, Lai WA et al. found that 46.1% of patients with actionable genetic alterations benefited from matched therapies, while Tsimberidou AM et al. reported improved outcomes, including longer PFS and extended OS, for patients with single genetic alterations who received matched therapies.^23,24^ Bonanno L et al. also suggested that NGS can effectively identify druggable alterations, leading to improved overall survival when matched with targeted therapies.^25^

## Supplementary Material

oyaf168_suppl_Supplementary_Material

## Data Availability

All data and materials supporting the conclusions of this article are available in the figures, tables, and Supplementary material, which are available to authorized users.
