# Supplementary material for: Utility and real-world clinical outcomes of next-generation sequencing in advanced non-small-cell lung cancer in the South Indian population
Source: Oncologist. 2025 Jul 31;30(7):oyaf168. doi: 10.1093/oncolo/oyaf168 (PMC12311926; doi:10.1093/oncolo/oyaf168)
Supplement: oyaf168_suppl_Supplementary_Material [file oyaf168_suppl_supplementary_material.docx]

Supplementary files


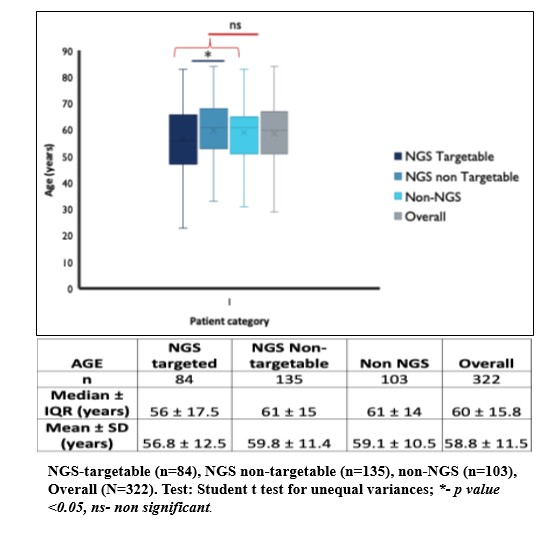


**Supplementary Fig-1: Age description in the study population across categories and overall distribution (yellow).**

**Supplementary Fig.-2: Distribution of gender across study population.**

**Supplementary Fig.-3: Distribution of smokers across study population.**

**Supplementary Fig.4: Distribution of PDL1 mutation (by category) in the study population.**


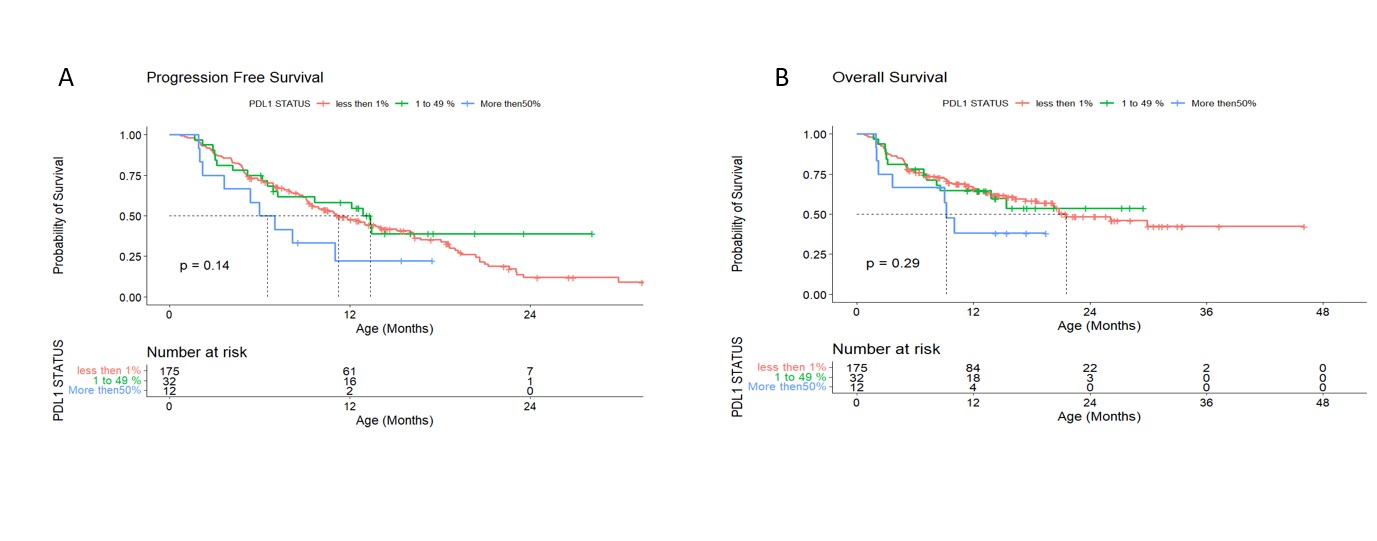


### **Supplementary Fig.5** Survival analysis of Kaplan–Meier curves depicting survival curves for all patients with PDL expression. Progression-free survival for all patients PDL1 expression (<1%, 1-49%, >50%) represented by A. OS for all patients PDL1 expression (<1%, 1-49%, >50%) represented by B.
